# Protocol for the CHORD project (community health outreach to reduce diabetes): a cluster-randomized community health worker trial to prevent diabetes

**DOI:** 10.1186/s12889-018-5419-4

**Published:** 2018-04-19

**Authors:** Nadia Islam, Thomas Gepts, Isaac Lief, Radhika Gore, Natalie Levy, Michael Tanner, Yixin Fang, Scott E. Sherman, Mark D. Schwartz

**Affiliations:** 1 NYU School of Medicine, Department of Population Health, New York, NY 10016 USA; 2 NYC Health + Hospitals, Bellevue Hospital, New York, NY USA; 3 NYU Langone Health, Department of Medicine, New York, NY USA; 4 New Jersey Institute of Technology, Department of Mathematical Sciences, Newark, NJ USA; 5 VA New York Harbor Health Care System, New York, NY USA

**Keywords:** Community health workers (CHWs), Prediabetes, Diabetes, Primary care, CHW integration, Chronic disease prevention, Community-clinic linkage, Patient-centered medical home

## Abstract

**Background:**

Type 2 diabetes mellitus (DM) affects 9.4% of US adults and children, while another 33.9% of Americans are at risk of DM. Health care institutions face many barriers to systematically delivering the preventive care needed to decrease DM incidence. Community health workers (CHWs) may, as frontline public health workers bridging clinic and community, help overcome these challenges. This paper presents the protocol for a pragmatic, cluster-randomized trial integrating CHWs into two primary care clinics to support DM prevention for at-risk patients.

**Methods:**

The trial will randomize 15 care teams, stratified by practice site (Bellevue Hospital and Manhattan VA), totaling 56 primary care physicians. The study cohort will consist of ~ 2000 patients who are 18–75 years of age, actively enrolled in a primary care team, able to speak English or Spanish, and have at least one glycosylated hemoglobin (HbA1c) result in the prediabetic range (5.7–6.4%) since 2012. Those with a current DM diagnosis or DM medication prescription (other than metformin) are ineligible. The intervention consists of four core activities – setting health goals, health education, activation for doctor’s appointments, and referrals to DM prevention programs – adjustable according to the patient’s needs and readiness. The primary outcome is DM incidence. Secondary outcomes include weight loss, HbA1C, and self-reported health behaviors. Clinical variables and health behaviors will be obtained through electronic medical records and surveys, respectively. Implementation outcomes, namely implementation fidelity and physicians’ perspectives about CHW integration into the clinic, will be assessed using interviews and CHW activity logs and analyzed for the influence of moderating organizational factors.

**Discussion:**

This is the first rigorous, pragmatic trial to test the effectiveness of integrating CHWs into primary care for DM prevention reaching a population-based sample. Our study’s limitations include language-based eligibility and the use of HbA1c as a measure of DM risk. It will measure both clinical and implementation outcomes and potentially broaden the evidence base for CHWs and patient-centered medical home implementation. Further, the intervention’s unique features, notably patient-level personalization and referral to existing programs, may offer a scalable model to benefit patients at-risk of DM.

**Trial Registration.**

Clinicaltrials.gov NCT03006666 (Received 12/27/2016).

## Background

Type 2 diabetes mellitus (DM) is a preventable chronic disease that affects 9.4% of US adults and children. It is estimated that 84.1 million American adults (33.9%) are prediabetic [1], and thus at risk of DM and subsequent cardiovascular disease. DM is a leading cause of death, one of the major causes of heart disease and stroke, and severely threatens quality of life. DM incidence can be reduced [2–8], even in the long term [9, 10], by identifying patients with prediabetes, half of whom may develop DM over the next decade [9], and by offering them proven behavior change interventions focusing on weight loss and physical activity.

Despite the potential for reduced morbidity and cost savings, primary care systems face several barriers to systematically deliver proven, preventive strategies to patients at highest risk of DM, as existing evidence has demonstrated that primary care teams have low levels of awareness and referral to evidence-based prevention programs [11]. In the traditional structure of visit-based encounters, barriers include a lack of time for clinical staff to conduct and maintain behavior change counseling, unclear delegation of non-visit based outreach tasks, and insufficient skills and tools for performing motivational and behavioral counseling interventions with populations of patients to optimize prevention of DM [12].

Community Health Workers (CHW) are frontline public health professionals who provide health coaching and social support within their patients’ communities [13]. Employing CHWs to conduct behavioral counseling, follow-up, referrals to programs, and education is a promising approach that may extend the capacity of health systems to better prevent and manage chronic conditions [14–18]. There is growing evidence that CHWs with appropriate training can serve as effective peer coaches in outpatient settings by performing motivational interviewing and other counseling techniques to promote change in health behaviors [19–22]. Culturally congruent social support is a key element of successful behavior change [23]. Thus, CHWs have been successful in counseling and motivating patients to engage in proven prevention strategies [24–27].

CHWs may offer a means of achieving the aims of the patient-centered medical home model (PCMH) and supporting primary care practices’ increasing efforts to work in an integrated manner to coordinate care for a panel of patients [14]. The addition of CHWs to the primary care team can improve care for patients with chronic disease at modest cost [15–17]. Moreover, CHWs integrated into primary care systems can improve preventive care and help to address social determinants of health (SDH) [13]. Investing in the delivery of DM prevention by CHWs in clinical settings may be a sustainable, scalable model for prevention, as evidenced by recent efforts to reimburse clinics for referral of prediabetic patients to YMCA-led Diabetes Prevention Programs (DPP) [18, 19].

Despite the potential for this growing workforce to enhance DM prevention efforts within the PCMH model, there is a need for high quality, randomized trials to assess specific models for CHW support; to aid with implementation of such model; and to identify effective strategies for recruitment, training, monitoring, and retention of lay personnel [16–18]. Here, we describe the protocol for a cluster-randomized study that aims to develop and test a model of CHW integration into primary care systems designed to prevent the onset of type 2 DM in a large population of underserved patients at risk. Our central premise is that CHWs are uniquely suited to engage patients, encourage lifestyle change through shared experiences and social support, and refer patients to community-based DM prevention resources, thus extending the reach of primary care (PC) beyond the clinic visit and reducing population risk for DM.

## Methods/design

### Study design

**Fig. 1:**
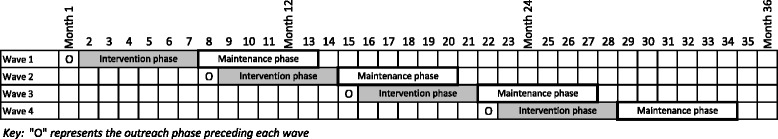
The trial will be conducted using a staggered entry design across four overlapping 12-month waves, each wave consisting of a 6-month intervention phase and a 6-month maintenance phase

This study is a pragmatic, cluster-randomized, controlled trial to evaluate whether CHW coaching within PCMHs is effective in preventing type 2 DM among patients with prediabetes. PC provider (PCP) teams will be randomized to the CHW intervention or to usual care (control) groups, stratified by the two hospital clinics in this study. Patients of providers in the intervention group will be eligible for CHW services, while patients of PCPs in the control group will not receive any CHW support. The 36-month intervention will be conducted using a staggered entry design across four, overlapping 12-month waves to account for CHW caseload limits. Each wave consists of a 6-month intensive phase of CHW-patient contact and a 6-month maintenance phase, with one month of patient outreach and recruitment preceding each wave (see Fig. 1).

### Setting

This study will take place in the primary care clinics of the Manhattan campus of the VA NY Harbor Healthcare System (VA) and Bellevue Hospital Center (BH), the flagship of New York City’s municipal hospital system, NYC Health and Hospitals. At the VA, there are 10 general PC teams and a women’s health team, each with 1 to 5 PCPs (total of 24), a nurse care manager, a licensed practical nurse, and a clerical associate, all caring for about 1200 patients per team. At BH, there are 4 PC teams, each with ~ 8 PCPs (total of 32), and 5 patient care associates, each caring for about 7000 patients. Teams at both sites share extended team members, including nurse care managers, mental health professionals, a social worker, a pharmacist, and a nutritionist.

### Study population

Eligible patients will be drawn from PCPs’ panels at the VA and BH. Patients are identified through the respective hospital system’s corporate data warehouses (CDW). Data from patients meeting eligibility criteria below will be extracted from the VA and BH CDW and merged into a secure, study database.

To qualify, patients must be 18–75 years of age, have at least one glycosylated hemoglobin (HbA1c) result in the prediabetic range (5.7–6.4%) in the 5 years prior to the start of the intervention, and be actively enrolled in a PCP’s panel (at least 1 PC visit in the past 2 years at the VA or at least 3 PC visits in the past 2 years at BH). Practice panels at BH are less stable; thus, 3 visits are required by patients at BH to establish a cohort likely to have longer-term follow-up in the practice. Patients must also speak English or Spanish, in order to communicate with bilingual CHWs. The main exclusion criterion is a prior DM diagnosis, defined as any ICD-9 or ICD-10 diagnostic code for DM applied during ambulatory encounters in the 2 years prior to the intervention, or any prescription of a DM medication, other than metformin alone, which can be used for DM prevention [28]. Lastly, prior to the intervention, PCPs may exclude any of their patients if they determine that they are not appropriate for the intervention for any reason. Sample sizes are detailed below.

### Study intervention

At each wave’s outset, ~ 250 patients will be randomly selected for outreach and paired with one of five CHWs (2 at BH, 2 at VA, 1 at both clinics). Patients are matched to a CHW according to the patient’s language and neighborhood of residence, as the study aims to cluster CHWs’ patients to facilitate CHW travel and expertise in local resources. Selected patients will receive a letter on behalf of their PCP alerting them of their DM risk status, available prevention resources, and introducing them to their assigned CHW. CHWs will then call patients to describe the program and seek verbal informed consent. Patients who decline participation will be asked if they are willing to be invited again in subsequent waves of the study. CHWs will continue outreach until they have met their caseload for the wave. This enrollment procedure and the following intervention have been approved by the appropriate institutional review boards at each study site (NYU: i16–00690, VA: MIRB 01609, BH: STUDY00000844).

**Fig. 2:**
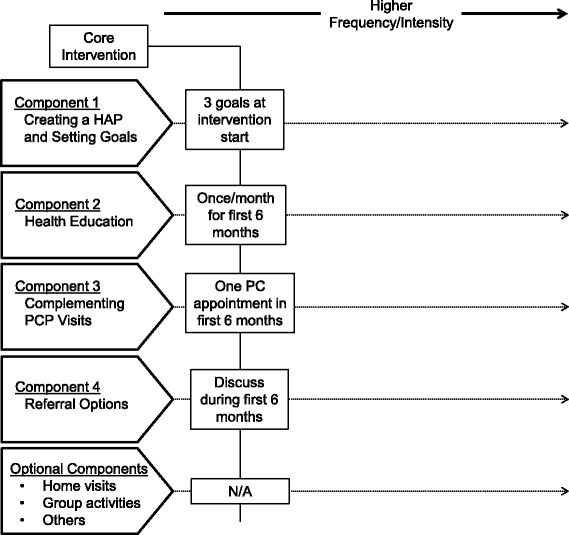
The intervention toolkit’s required and optional components and their respective minimum intensity levels

If the patient consents, the CHW will work with the patient to complete a baseline intake survey and to create individualized and evidence-based goals that will be translated into a health action plan (HAP). The HAP will reflect the patient’s activation level (defined below) and preferences, as there is evidence that the fit between an intervention and a patient’s motivations, beliefs, and perceptions encourages activation, goal-setting, and ultimately behavior change [29, 30]. The HAP will be built from a toolkit consisting of four categories of activities detailed below. This toolkit standardizes the intervention across CHWs and focuses on evidence-based strategies for preventing DM. When selecting strategies for the HAP, CHWs counsel all patients to engage in the most intensive prevention activities that they will accept. The highest intensity activities include the most frequent possible CHW contact and referral to the 16-week Diabetes Prevention Program (described below) and/or other existing hospital- and community-based DM prevention programs. Figure 2 illustrates the toolkit’s standard elements and how CHWs and patients may personalize the intervention. However, to ensure that all intervention patients receive a baseline exposure to the CHW program, all patients, irrespective of activation, will receive the core intervention, composed of low intensity settings of the four toolkit components:

#### Creating the HAP and setting goals

The HAP will be created collaboratively by the patient and CHW and reviewed by the research team at a weekly case conference. The CHW returns to the patient with the revised plan to make any final changes. The HAP specifies the patient’s goals and how he/she will engage with the intervention activities. The CHW guides the patient in setting well-defined and achievable goals. CHWs will use motivational interviewing techniques when revisiting these goals with patients during the intervention. The HAP, along with 3-month and 6-month progress updates during the intensive phase, will be shared with the patient’s PCP, who may suggest modifications based on their knowledge and relationship with the patients.

#### Health education

CHWs will conduct one-on-one health education sessions on topics including health self-management, exercise, and healthy eating. CHWs will conduct at least one session a month during the 6-month intensive period, but activated patients may request supplementary modules and have more frequent contacts, as often as weekly. Sessions will occur by phone or in person according to patient preference. CHWs will attempt to meet each patient in person at least once during the intervention, at either the patients’ home, neighborhood, or clinic. Patients also will receive a packet of educational materials about DM, and exercise and nutrition to prevent DM, written at less than an 8th grade reading level in English or Spanish, containing material aligned with each monthly health education session.

#### Complementing PCP visits

CHWs will call patients before and/or after PCP visits, in order to encourage discussion about diabetes prevention with the doctor or to review the doctor’s suggestions. CHWs are available to meet patients at clinical sites for in-person conversations.

#### Referral to hospital- and community-based DM prevention programs

CHWs will counsel patients about DM prevention programs offered at the patients’ hospital or in their community, making referrals if the patient would like to participate. Both the VA and BH have weight loss clinics open to all patients (VA: *MOVE!*; and BH: Healthy Lifestyles). Further, the YMCA and New York City Department of Health and Mental Hygiene hold DPP classes offered at discounted cost that will be paid for by the study for intervention patients. CHWs will guide patients through this network of DM prevention programs and other local resources, such as farmers markets, community-based organizations, and hospital social work offices.

The intervention toolkit allows for personalization of this core intervention in two ways. First, it contains additional activities CHWs may offer to patients, including home visits and an ongoing schedule of group activities such as neighborhood walks or group grocery shopping. Second, a patient may intensify any activity by increasing their engagement in core intervention components. For instance, patients may request more frequent calls with the CHW, additional health education modules, or enroll in multiple hospital- or community-based programs. Hospital regulations permitting, patients may also text-message with their CHW, reinforcing the social support dimension of the intervention. The toolkit balances delivering a uniform, evidence-based DM prevention intervention with patient-level tailoring expected to increase sustained behavioral change [29, 30]. Standardized protocols will be used to ensure that CHWs dedicate sufficient time to engaging patients with lower activation levels.

Following the 6-month intensive phase, the intervention transitions into a 6-month maintenance phase, during which CHWs will conduct monthly check-in phone calls with their patients; follow-up in-person visits or health education will not be offered during this phase. Patients may also contact their CHW for support. Beginning during the first wave’s maintenance phase, we will recruit patients that have completed the intensive intervention phase as “buddies” that can provide additional social support for new intervention patients in subsequent waves.

### Study outcomes

#### Patient outcomes

The primary outcome of the study is type 2 DM incidence. Any patient with at least two encounters with a DM diagnosis (ICD-9 codes 250, 357.2, 362.0, or 366.41) will be defined as having DM. Each incident case will be verified by looking for evidence of hyperglycemia (at least two fasting glucose readings > 126 mg/dL or an HbA1c ≥6.5%), or the prescription of DM medications, to avoid under- or overestimating DM incidence. A similar approach was used by Miller and colleagues in the VA DM Epidemiology Cohort [28]. Both change in weight in pounds (and body mass index, BMI) and the proportion of overweight (BMI 25.0–29.9) and obese (BMI ≥ 30) patients are measured as secondary outcomes over the course of the study. A range of additional clinical outcomes will be measured – glycemic control (HbA1C), blood pressure, smoking status, and Framingham Risk Score (10-year) – all derived from the respective  CDWs at each hospital practice throughout the study. Health service utilization – number of PC visits, PC telephone encounters, emergency and urgent care visits, visits to hospital-based wellness programs, and visits to the nutritionist – will also be assessed at baseline and conclusion and will be drawn from the medical record. Self-reported health attitudes and behaviors, including health self-perception, diet, exercise, and activation are further patient-centered outcomes, measured in the patient intake survey (see Table 1).

**Table 1:** Patient-centered outcomes by source, domain, and measurement frequency over the course of one wave.^a^ *EMR* Electronic medical record; Intake refers to intake interview by community health worker

Source^a^	Domain	Baseline	Quarterly	1-Year
Socio-demographic				
EMR	Sex, age, race, language	✓		
Intake	Marital status, education, residential status, employment status, income	✓		
	Social determinants of health	✓		
Health Attitudes and Behavior				
Intake	Self-reported health status	✓		✓
	Diabetes risk belief	✓		✓
	Prediabetes awareness	✓		✓
	Physical activity	✓		✓
	Diet	✓		✓
	Awareness of prevention resources	✓		✓
Patient Activation				
Intake	6-item Patient Activation Measure ®	✓		✓
Clinical Data				
EMR	Weight	✓	✓	✓
	BMI	✓	✓	✓
	Hemoglobin A1C	✓	✓	✓
	Serum glucose	✓	✓	✓
	Blood pressure (Systolic/Diastolic)	✓	✓	✓
	Total cholesterol	✓	✓	✓
	LDL cholesterol	✓	✓	✓
	HDL cholesterol	✓	✓	✓
	Triglycerides	✓	✓	✓
	Smoking status	✓	✓	✓
Health service utilization: # of				
EMR	Primary care encounters	✓	✓	✓
	Walk-in clinic encounters	✓	✓	✓
	Emergency Department encounters	✓	✓	✓
	Hospitalizations	✓	✓	✓
	Hospital weight loss clinic	✓	✓	✓
	Nutrition encounters	✓	✓	✓
Diagnostic Codes for:				
EMR	Type 2 Diabetes	✓	✓	✓
	Hypertension	✓	✓	✓
	Chronic Kidney Disease	✓	✓	✓
	Cardiovascular Disease	✓	✓	✓
	Peripheral Artery Disease	✓	✓	✓
	Obesity	✓	✓	✓

#### Implementation outcomes

Implementation fidelity and PCPs’ perspectives about the CHW intervention and CHW integration into the clinic for DM prevention will be assessed (see Table 2) considering factors theorized to influence implementation outcomes, including characteristics of the intervention; of PCPs, primary care teams, and CHWs; and of the community and policy context that shapes health care access and resources in the city [31].

**Table 2:** Implementation outcomes, by source, domain, and measurement frequency

Source	Domain	Baseline	Semi-annually	Annually
Implementation Fidelity				
CHW Assessments & Intervention Tracking	Content of CHW activities		✓	
	Patient coverage		✓	
	Duration of patient follow-up		✓	
	Frequency of patient contact		✓	
Perceived Acceptability, Adoption, Appropriateness, & Feasibility of CHW Integration				
Surveys & Interviews	Approaches to management of pre-DM patients, beliefs about CHWs and working with CHWs to prevent DM, awareness and use of DM prevention resources in PC practice and in the community	Interviews with subset of all PCPs. Survey of all PCPs	Interviews with subset of intervention PCPs, PC team staff and CHWs	Interviews with practice administrators. Survey of all PCPs at intervention-end
	Changes to PC team function managing pre-DM patients		Interviews with subset of intervention PCPs, PC team staff and CHWs	

Implementation fidelity will be assessed by the content of CHW activities and the coverage (number of patients reached), duration (how long each patient is followed up), and frequency (how often patients are contacted) of CHW-patient contact. The perceived acceptability (perception that the intervention is agreeable or satisfactory); adoption (intention to use or deploy the intervention); appropriateness (perception about the fit or relevance of the intervention for a practice setting); and feasibility (perception about the extent to which the intervention can be successfully used or carried out within a given setting) of the CHW intervention and CHW integration into the clinic will be assessed using surveys of PCPs and interviews with PCPs, clinical staff, CHWs, and practice administrators at each site [32]. Exposure to the CHW intervention may affect how providers individually and PC teams collectively function in preventing DM and working with a CHW, thus potentially altering their usual practices in managing prediabetic patients.

A survey given to all PCPs at baseline and intervention-end and semi-structured interviews every 6 months with a subsample of PCPs and clinical staff in the intervention group are used to assess their attitudes and approaches regarding DM prevention and incorporating peer coaching into clinical care. Semi-structured interviews will be conducted annually with practice administrators at BH and the VA selected as key informants for their expertise in DM prevention and their clinic’s administrative structure.

Through weekly activity logs, CHWs track their patient contacts and case notes, and the researchers will conduct interviews with the CHWs every 6 months to explore challenges, successful and unsuccessful strategies, and their perspective on their role as part of the clinical team.

### Data collection

#### Intervention outcomes: Patient data collection

As a pragmatic, population-based trial, this study aims to understand the impact of our interventions on panels of patients in a real clinic environment and therefore will not collect any clinical data directly from patients. Instead, the study uses measurements taken during regular clinic visits and recorded in the EMR. The study team works directly with data analysts at the VA and BH to extract the study variables from the EMR and to maintain the study database.

Prior to the initial patient contact, CHWs will receive basic demographic and clinical data, drawn from the EMR, for their patient panel. During the initial contacts with patients, CHWs will conduct a comprehensive intake process. The intake consists of demographic background; self-reported health status, physical activity, and diet; DM risk perception; prediabetes awareness; previous use of prevention resources; social determinants of health; and the 6-item Patient Activation Measure (PAM-6) [33]. Intervention group patients complete this survey at baseline, as part of the intake assessment, and at the end of their 1-year intervention period. Control patients will receive, by mail, a self-administered version of the survey only at the close of the 1-year intervention period. As a response incentive, patients will receive $10 gift cards upon completing the survey.

#### Implementation outcomes: Providers, clinical staff, and CHWs

In addition to patient enrollment in the intervention, PCPs in all primary care teams (*N* = 56; 32 at BH and 24 at VA) will be enrolled as study participants in order to assess barriers and facilitators to implementation. A survey will be given to all PCPs, in intervention and control arms, at baseline and intervention close. A subsample of key informants (N = ~ 8–10 at each site) will be interviewed, including PCPs and clinical staff (semiannually) and administrators (annually). CHWs will also be interviewed every 6 months to assess challenges, enabling factors, and best practices in working with prediabetic patients. All interviews will be audio recorded and transcribed for analysis along with field notes recorded by the interviewer.

Data to assess implementation fidelity will be collected across each study phase through a variety of measures. CHWs complete standardized reports on their patient interactions in their weekly activity logs, including the number of patients they target, reach, and refer, and the number, type, and length of patient interactions. The rate of patient contact failure will also be tracked by recording returned mail and out-of-service or incorrect phone numbers. Meeting minutes will also be recorded from weekly CHW-supervisor meetings and will document activities, challenges, and lessons learned by CHWs. Lastly, every month, the CHW Coordinator will monitor one of each of the CHWs telephone counseling sessions to ensure counseling skills, offer feedback, and ensure treatment fidelity. The calls will be evaluated using a standardized checklist.

### Data analysis

#### Sample size and statistical power

**Fig. 3:**
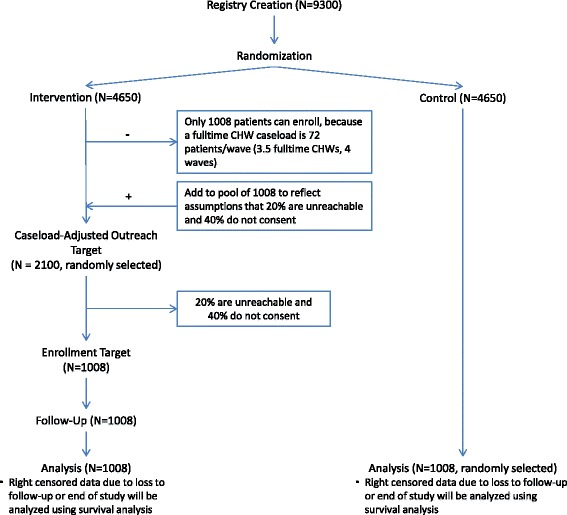
Study design flowchart

Analyses will be conducted at the patient level, aggregated to their PCP, controlling for the cluster-randomized design. The annual hazard rate of incident cases of DM over time is the primary clinical outcome for power calculations. The current rate of HbA1c prediabetes screening among eligible patients without prevalent DM since 2012 is 73–74% and is likely to approach 80% due to clinical guidelines and increased healthcare systems transformation efforts. Of those with an HbA1c test since 2012, 41–44% of patients fall within the prediabetic range. While the above inclusion and exclusion criteria return 9300 eligible patients, 8200 at BH and 1100 at the VA, CHW caseload limitations will prevent us from reaching all eligible patients during the 3-year intervention. Assuming a caseload of 72 patients per fulltime CHW per wave (with 3.5 fulltime equivalent CHWs across both sites), an estimated 20% unreachable rate, and a 60% consent rate among intervention patients, our total intervention sample is anticipated to be 1008 patients, split evenly at each site (see Fig. 3). Control patients will be randomly selected for each wave at each site. In the prediabetes population, the 3-year DM incidence is estimated to be 15% (based on preliminary analysis of a similar NY VA cohort from 2010 to 2012, followed through 2015). A conservative estimate of the intracluster correlation coefficient (ICC) is 0.01, based on the median value found in a systematic review of cluster randomized trials [34] and the highest value calculated in our previous VA study [35]. This planned sample size will provide at least 80% power to detect an expected difference in annual hazard rates of time to DM incidence between two arms (3.5% per year in intervention vs. 5.4% per year in control), at a 0.05 significance level. By assuming exponential distribution, this difference in annual hazard rates can be approximated by the difference in incidence rates over 3 years (10% in intervention vs. 15% in control).

#### Quantitative analysis

We will conduct both an intention-to-treat analysis (all patients randomized to either study group, including those who decline to participate or we cannot contact) and a per protocol analysis (only patients who engage in the intervention according to the protocol). Descriptive analysis techniques (measures of central tendency and variability, frequencies, and proportions) will be conducted to describe baseline characteristics of participants (patients and their PCMH staff) in the control and intervention groups. Chi-square tests and t-tests will be used to check if these baseline characteristics are balanced between the two study groups. For continuous outcomes, normal assumptions will be checked, and non-parametric alternatives and transformations will be considered as needed.

To assess the overall effectiveness of the CHW intervention, we will compare cumulative DM incidence rates and time to DM incidence (in addition to other outcomes: weight loss, obesity rate, and health care utilization) between the two study groups, using intention-to-treat and per protocol analyses. DM cumulative incidence rates at 12-month follow-up of the intervention and control arms, aggregated across the 4 study waves, will be compared using a chi-square test. Similar analyses are conducted for other categorical outcomes (e.g. obesity rate) and t-tests will be used to compare continuous outcomes (e.g. weight change). We will further conduct generalized linear mixed-effects modeling [36] for these outcomes, which (1) takes into account the correlation among patients within teams, (2) analyzes repeated measures at several time-points (baseline, midpoint, and post-intervention) simultaneously, (3) includes team level or patient level baseline covariates that are found to be unbalanced between two arms, and (4) deals with attrition and other missing data.

We will use multilevel survival analysis to determine the effect of the intervention on time to DM, as patients are enrolled in waves and the time to DM incidence may be censored by the end of study. Specifically, we will fit Cox proportional hazard regression models with mixed effects [37] to examine the hazard ratio between two groups, adjusting for important covariates and taking into account the correlation among patients within teams.

Similar generalized linear mixed-effects models will be used for the secondary outcomes, to explore the relationship of the intervention’s effectiveness to specific patient and provider or team characteristics. The study investigates interactions between the intervention and the patients’ level of activation, co-morbidities (hypertension, depression, and smoking), health care utilization, and perceptions of the quality of care.

Secondary analyses to better understand the impact of the individual components of this complex set of interventions will be conducted. Since the intervention is designed to test the effectiveness of an overall strategy, the study seeks to determine which of the intervention elements and dose of intervention (e.g., number of CHW contacts, number of mailings, or successful referral to hospital- or community-based programs) are most strongly associated with any improved outcomes. Subgroup analyses will also be conducted, to see which subgroups may benefit more from the intervention than the others. Such subgroup analyses should be considered as exploratory analyses and any resulting findings should be confirmed by independent studies.

To limit bias due to contamination and missing values, the baseline characteristics of patients who switch teams or are removed from the panel or have incomplete repeated outcomes will be assessed and compared with those of the other participants. The (generalized) linear mixed-effect models discussed earlier can deal with attribution problems and other missing data, under the missing-at-random assumption [38]. To better understand the missing mechanism, outcome variables will be summarized by discontinuation status in each group. If the trajectories of the primary outcome variables from those with incomplete data are similar to those with complete information, the data may be missing at random; otherwise, they may be missing not at random. A sensitivity analysis will then be conducted to assess the impact of missing not-at-random data on our analysis. The sensitivity analysis will test whether our primary statistical analysis remains credible if it includes cases where those patients with incomplete data have outcomes that are unfavorable to the intervention group of the study. The sensitivity analysis will be conducted via multiple imputation using sequential modeling [39].

Survey data will be used to assess PCPs’ readiness to work with CHWs for DM prevention. Baseline and post-intervention scores will be compared using Wilcoxon signed-rank tests. To assess implementation fidelity, baseline and 6-monthly scores on the coverage, duration, and frequency of CHW-patient contact for BH and VA patients will be compared using ANCOVA. Content of delivery will be assessed qualitatively based on a sample of CHW-patient contacts. Implementation fidelity will be compared across BH and the VA to examine if differences in organizational setting shape the implementation process.

#### Qualitative analysis

All interview transcripts and field notes will be analyzed for common themes by two independent researchers using both deductive and inductive approaches. First, initial codes will be identified based on our study aims and on relevant prior implementation research. Two researchers will independently read the transcripts, identifying new codes based on any new themes or subthemes that emerge from the data. Second, the researchers will meet with each other and the study research team to compare and discuss codes, resolve discrepancies, and iteratively develop a codebook with code names and meanings. Third, researchers will apply the codebook to the transcripts. Researchers will identify patterns, common themes, and connections among themes throughout the transcripts. Baseline interviews will be used to refine the intervention toolkit and to explore anticipated barriers and facilitators to incorporating CHWs into the PC teams’ workflow. Mid- and post-intervention interview and case review data will help us to determine key barriers, facilitators and lessons learned from staff and CHWs regarding the implementation of CHW support and the integration of CHWs in PC practice for DM prevention in an underserved population, accounting for organizational setting.

## Discussion

The study has several limitations. First, study enrollment is limited to English and Spanish speakers. Second, several studies have discussed the limitations of using HbA1c as a measure of diabetes risk, noting the measure likely under-estimates risk for diabetes [40–45].

Despite these limitations, the study has noteworthy strengths. To our knowledge, our trial is the first to use a rigorous, pragmatic trial design to test the effectiveness of integrating CHWs into primary care teams for DM prevention. Though CHWs will not be able to reach all eligible patients, the trial anticipates reaching over 1000 intervention group patients across the study years. Second, our study assesses both clinical outcomes and implementation outcomes, such as provider attitudes and intervention fidelity, which can inform the sustainability and scalability of the intervention.

A growing literature suggests that CHWs – by integrating with primary care teams to offer targeted support to patients with greater risks and gaps in care – can improve health outcomes and increase population-level reach. Still, there is limited information on best practices, workflows, and procedures for implementation to maximize success. Our study findings will enhance PCMH efforts by incorporating non-clinical staff into primary care, promoting patient activation and targeting specific interventions to increase patient motivation and efficacy in making healthy changes to prevent DM. Finally, unique features of the study are the personalization of CHW services according to patient activation level, and referral to evidence-based DM prevention resources. By offering an individualized health coaching intervention in conjunction with referrals to “gold-standard” DM prevention efforts like the DPP, our study provides a framework for integrating personalized behavior change efforts with broad-scale, evidence-based programs.

The current health care context affords tremendous opportunities to implement sustainable models of CHW integration into health care systems. The proposed study will provide important evidence for the implementation of such efforts in safety-net systems addressing a critical public health prevention challenge.

## Abbreviations

BH: Bellevue Hospital
BMI: Body Mass Index
CDW: Corporate Data Warehouse
CHW: Community Health Worker
DM: Diabetes Mellitus
DPP: Diabetes Prevention Program
EMR: Electronic Medical Record
HAP: Health Action Plan
HbA1C: glycosylated hemoglobin
PAM®-6: 6-item Patient Activation Measure®
PC: Primary Care
PCMH: Patient Centered Medical Home
PCP: Primary Care Physician/Provider
SDH: Social Determinants of Health
VA: VA New York Harbor Healthcare System
